# Therapeutic Efficacy of Ultrasound-Guided High-Voltage Long-Duration Pulsed Radiofrequency for Pudendal Neuralgia

**DOI:** 10.1155/2021/9961145

**Published:** 2021-07-30

**Authors:** Feng Ji, Shuzhuan Zhou, Caixia Li, Yongyan Zhang, Hua Xu

**Affiliations:** ^1^Department of Anesthesiology, Yueyang Hospital of Integrated Traditional Chinese and Western Medicine Affiliated to Shanghai University of Traditional Chinese Medicine, Shanghai 200437, China; ^2^Department of Anesthesiology, Marine Corps Hospital of PLA, Chaozhou 521000, Guangdong Province, China

## Abstract

Pudendal neuralgia (PN) is a complex disease with various clinical characteristics, and there is no treatment showing definite effectiveness. This study is aimed at evaluating the clinical efficacy of ultrasound-guided high-voltage long-duration pulsed radiofrequency (PRF) for PN. Two cadavers (one male, one female) were dissected to provide evidence for localization of the pudendal nerve. Patients diagnosed as PN who failed or were intolerant in regular medication were screened for diagnostic local anesthesia block of the pudendal nerve before recruitment. Twenty PN patients were enrolled in this study. In the PRF procedure, the needle tip was inserted medially into the internal pudendal artery under ultrasound guidance. The position of the PRF needle tip was then adjusted by the response of the pudendal nerve to the electrical stimulation within the pudendal area (42°C, a series of 2 Hz, and 20 ms width pulses that lasted for 900 s). Alleviation of pain was assessed by the visual analogue scale (VAS) and sitting time pretreatment and on 7 d, 14 d, 1 m, 2 m, 3 m, and 6 m posttreatment in outpatient follow-up or by telephone interview. Two patients were lost due to intervention-irrelevant reasons. Patients showed significantly decreased VAS scores on 7 d after RFP, compared with pretreatment status (7.0 ± 0.9 vs. 3.2 ± 1.7, *P* < 0.001). The efficacy remained steady till the end of 6 months, with a final remission rate of 88.9%. Sitting time also significantly lengthened following PRF (7 d, 14 d, 1 m, 2 m, 3 m, and 6 m vs. pretreatment, all *P* < 0.05). Only short-term ipsilateral involuntary convulsion of the lower extremity was reported in one patient, who recovered within 12 h. Six patients were treated with nonsteroidal drugs for a short time. All patients stopped taking medication finally. In conclusion, the ultrasound-guided high-voltage long-duration PRF approach not only reduced the pelvic pain caused by PN but also improved the quality of life by extending sitting time without nerve injury.

## 1. Introduction

Pudendal neuralgia (PN) refers to neuropathic pain in the pudendal nerve innervation region, which may occur in the entire perineal region or in one of its branches, and is often accompanied by symptoms such as rectal and anal foreign body sensation, distension, frequency and urgency of urination, and sexual dysfunction [1, 2]. According to the survey by the International Pudendal Neuropathy Association, the incidence of PN in the general population is about 1/100,000 [3]. However, Spinosa et al. [4] documented the incidence at 1% in the general population, which is higher in females than in males, with more unilateral cases [3]. PN patients present a variety of clinical symptoms and usually need multidisciplinary treatment. In addition, due to the pain in private parts, patients tend to have a longer course of disease and more severe symptoms before willing to visit the doctor, which would seriously affect their quality of life. However, there is still a lack of definite treatment because of its unclear pathogenesis.

Radiofrequency has been used to treat pain for nearly a century. It is widely applied to regions including the head, neck, chest, waist, and sacral region [5–9]. However, with the extensive application of continuous radiofrequency treatment, clinicians found that the heat or improper operation would cause unacceptable compilations due to permanent nerve injury, such as continuous numbness and fecal incontinence [10]. Since the emergence of the pulsed radiofrequency (PRF) technique, these situations can be mostly avoided because of its lower temperature (no more than 50°C) during treatment [11, 12]. It has been increasingly used in the treatment of chronic pain, including PN. Studies reported that CT-guided pudendal nerve PRF treatment through the sacrotuberous ligament puncture to the pudendal canal could obviously relieve the symptoms of PN [13, 14]. CT-guided high-voltage long-duration PRF treatment was also applied to reduce the pain in patients with postherpetic neuralgia, as well as PN [15, 16]. However, radiation in CT scan may limit its further clinical application.

Compared with other image-guided technology, ultrasound is playing an increasingly important role in pain diagnosis and treatment due to its advantages of radiation-free, convenient, and real-time positioning guidance [17–19]. Ultrasound-guided PRF treatment at the ischial spine was reported effective for PN [18]. Its clinical efficacy and safety have also been confirmed by a randomized controlled clinical study [19]. However, the application of ultrasound-guided high-voltage long-duration PRF in the treatment of PN has not been reported yet. In the present study, we investigated the anatomical position of the pudendal nerve at the transverse section of the ischial spine and its adjacent relationship with the surrounding tissue. Eligible patients were then selected to receive ultrasound-guided high-voltage long-duration PRF treatment at the ischial spine. Its clinical efficacy and especially improvement in the quality of life were observed. It may provide a new option for PN treatment.

## 2. Materials and Methods

### 2.1. Anatomical Study

Two adult cadavers (one male and one female) formalin embalmed and fixed were used (provided by the Human Anatomy Teaching and Research Department of the Basic Department of Naval Military Medical University). The cadaver specimens had intact pelvis and pelvic organs. The subcutaneous tissues and muscles of the buttock were opened layer by layer to expose the subgluteal space, the sacrospinous ligament, and its adjacent vessels and nerves. The anatomical relationship between the sacrospinous ligament and the pudendal nerve was confirmed.

### 2.2. Patients

Patients diagnosed as PN who received treatment were recruited from the pain center of Changhai Hospital from Sep 1, 2015, to Oct 31, 2016. The PN diagnosis was based on the Nantes criteria (Table 1) [1]. They were unresponsive to medication therapy (12 patients) or intolerant to the side effects (8 patients) at recruitment. This study was approved by the Ethics Committee of Changhai Hospital Affiliated to the Naval Military Medical University.

Inclusion criteria are as follows: (1) aged from 18 to 80 years old and no sex limitation; (2) patients who were not satisfied with conservative treatment or intolerant to the side effects; and (3) be able to sign an informed consent form. Exclusion criteria are as follows: (1) patients with pelvic organic disease that may also cause pain in the pudendal region; (2) patients with malignant or autoimmune diseases that cause pain; (3) pregnant women; (4) patients with any coagulation disorder; and (5) patients who are unable to complete the outpatient or telephone interview.

### 2.3. Diagnostic Block of the Pudendal Nerve

**Figure 1:**
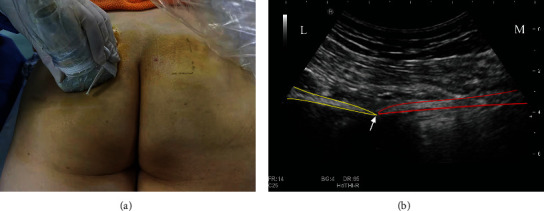
Diagnostic block of the pudendal nerve under ultrasound guidance. (a) Position and direction of the ultrasonic probe. (b) Transverse section of the ultrasound image in the ischial spine. Red outline: sacrospinous ligament; yellow outline: ischium; white arrow: ischial spine. L: lateral; M: medial.

**Figure 2:**
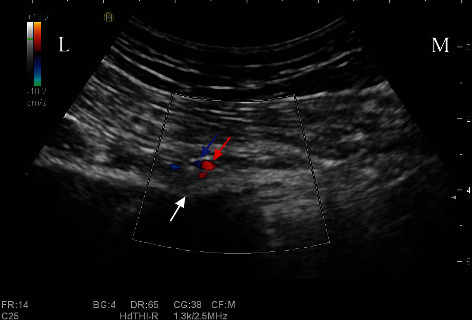
Internal pudendal artery presented in color Doppler. White arrow: ischium spine; red arrow: internal pudendal artery; blue arrow: internal pudendal vein. L: lateral; M: medial.

Effective pudendal nerve block is an essential approach for the inclusion of patients. The patient was in the prone position, and a low-frequency curved-array probe (C251/1~5 MHz, Hitachi Noblus, Japan) was used to scan from the posterior superior iliac spine downwards to the transverse section of the ischial spine (Figures 1(a) and 1(b)). The internal pudendal artery above the ischial spine was identified in color Doppler mode (Hitachi Noblus, Japan) (Figure 2). Outside of the ultrasound probe, a nerve block needle (Pajunk, 21 G∗100 m, Germany) penetrates the skin near the internal pudendal artery by the in-plane technique (the pudendal nerve mostly located in the medial part of the internal pudendal artery). The nerve stimulator (Braun Stimuplex, Germany) was applied to detect movement of the patient's pain site, and the needle tip position was adjusted accordingly. When the current is less than 0.4 mA and the pain site movement was induced, an injection of 5 ml 1% lidocaine was applied with 10 min observation. In bilateral cases, diagnostic block should be applied to bilateral pudendal nerves. Numbness and pain relief of more than half indicated the effectiveness of the diagnostic block and eligibility for recruitment of PRF treatment.

### 2.4. PRF Procedure

**Figure 3:**
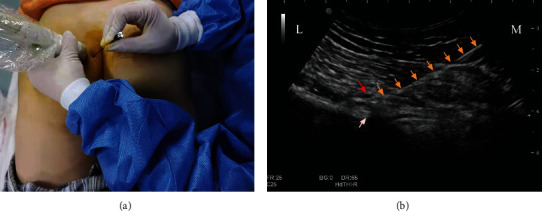
Procedure of pulsed radiofrequency. (a) The relationship between the location of the PRF needle and the position of the ultrasonic probe. (b) The track of the PRF needle and the position of the needle tip. Orange arrow: radiofrequency puncture needle; red arrow: internal pudendal artery; white arrow: ischium spine. L: lateral; M: medial.

In the PRF procedure, the patient was placed in the prone position on a sterilized sheet with routine disinfection. A 20 G radiofrequency needle (Cosman) was used for puncture, and the puncture process was the same as the diagnostic block of PN (Figures 3(a) and 3(b)).

#### 2.4.1. Test Parameters

In the sensory test (50 Hz), when the voltage was 0.3-0.5 V, the patient could feel the tingling sensation at the pain site, which indicated that the puncture needle tip was around the pudendal nerve. When the voltage was higher than 2 V in the motor test (2 Hz), the lower limb movement of the same side was not induced, indicating that the puncture needle tip was far from the sciatic nerve. During PRF, the radiofrequency instrument was set as the manual pulse treatment mode, and the RF parameters were set as follows: temperature 42°C, stimulation frequency 2 Hz, pulse width 20 ms, and duration 900 s; the field intensity began at 40 V and gradually increased until the patient has an intolerable abnormal sensation (such as burning sensation) in the pain site (the intensity was not more than 80 V to avoid nerve damage caused by high temperature). Patients with bilateral pain were treated with bilateral PRF. The procedure was performed with the radiofrequency therapeutic apparatus (Baylis, Canada).

### 2.5. Outcome Measures

Through outpatient or telephone follow-up, the pain intensity of patients was evaluated before intervention and on 7 d, 14 d, 1 month, 2 months, 3 months, and 6 months after high-voltage long-duration PRF treatment. The visual analogue scale (VAS) was applied for pain assessment, with 0 indicating no pain, 1-3 mild pain, 4-6 moderate pain, and above 7 severe pain. The patients' maximum sitting time before the onset of pain (i.e., sitting time) was evaluated according to the assessment method and criteria of previous studies [20, 21]. Briefly, the assessment was performed in the afternoon, and the patient was asked to sit in a comfortable position while the sitting time was recorded as the maximum time when the patient reported being too painful to keep on sitting. The sitting time of patients before treatment and on 7 d, 14 d, 1 month, 2 months, 3 months, and 6 months after treatment was evaluated in the outpatient follow-up by the same physician. However, if the patients were unable to come for the outpatient follow-up, self-assessed results were obtained through telephone interview under the guidance of the same physician instead (Supplement Table 3).

### 2.6. Statistical Analysis

The measurement data were expressed in terms of mean ± standard deviation (*X* ± SD). Repeated-measures one-way ANOVA was used for within-group comparison and Bonferroni's test for between-group comparison. *P* < 0.05 was considered statistically significant. SPSS 22.0 software was used in the statistical analysis.

## 3. Results

### 3.1. Anatomical Study

Two cadaver specimens (embalmed) were examined, including one Asian man (death age 76, height 172 cm, and BMI 25.7 kg/m^2^) and one Asian woman (death age 86, height 158 cm, and BMI 19.2 kg/m^2^).

**Figure 4:**
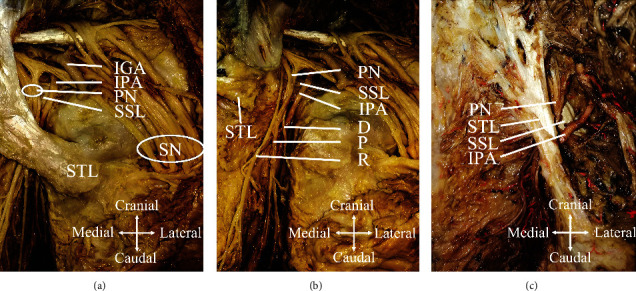
Anatomical study of the pudendal nerve in cadavers. (a) Pudendal nerve and its adjacent tissues. (b) Pudendal nerve and its branches of a male cadaver (dissection of the sacrotuberous ligament). (c) Pudendal nerve and its adjacent tissue in a female cadaver. STL: sacrotuberous ligament; SSL: sacrospinous ligament; IPA: internal pudendal artery; IGA: inferior gluteal artery; PN: pudendal nerve; SN: sciatic nerve; D: dorsal nerve of the penis; P: perineal nerve; R: inferior rectal nerve.

As indicated by a previous study [22], the course of bilateral pudendal nerves in two specimens was between the lesser sciatic foramen composed of the sacrospinal ligament and sacrospinal ligament. The pudendal nerve was located on the surface of the sacrospinal ligament, medially to the sciatic spine and adjacent to the internal pudendal artery (Figure 4).

At the cross of the sciatic spine in the male cadaver, the pudendal nerve was 13 mm medial to the sciatic spine, and the internal pudendal artery was 6 mm lateral to the pudendal nerve. In the female cadaver, the pudendal nerve was 11 mm medial to the sciatic spine, and the internal pudendal artery was 6 mm lateral to the pudendal nerve (Table 2). Anatomical results provided a position landmark for ultrasound-guided PRF at the ischial spine.

### 3.2. Patients

A total of twenty patients were finally enrolled, including six males and fourteen females, aged 42~68 yrs (57 ± 4.7 yrs) (Table 3, Supplement Table 1). Seven patients reported pain in the genitals and five in the anus, while the rest (eight) covering the anus, genitals, and perineum. Twelve patients reported unilateral pain, and eight patients were bilaterally involved. Patients were informed of the risks, and a written informed consent form was provided. Eighteen patients completed the 6-month postoperative follow-up, and two patients were lost because they could not be contacted by phone or were unwilling to participate in outpatient follow-up after treatment. Finally, sixteen patients stopped taking medication immediately after PRF. Four patients still reported short-term pain in the initial stage after PRF, took tramadol+pregabalin intermittently for a short time, and stopped taking medication at final visits.

### 3.3. VAS Score

**Figure 5:**
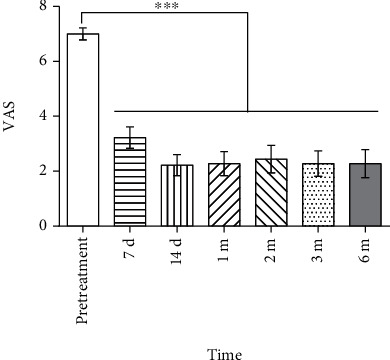
Results of the visual analogue scale (VAS) before and after high-voltage long-duration pulsed radiofrequency (PRF) treatment. VAS significantly decreased on 7 d after treatment and remained steady till 6 months. There was no significant difference among all the time points after treatment (pretreatment vs. 7 d, 14 d, 1 m, 2 m, 3 m, and 6 m posttreatment; ^∗∗∗^*P* < 0.001).

All the patients reported moderate or severe pain, with VAS scores of 7.0 ± 0.9 points before treatment. The VAS score on day 7 after the treatment (3.2 ± 1.7) was significantly lower than that before treatment (*P* < 0.05). The VAS score did not significantly change since day 14 after treatment (all *P* > 0.05, Figure 5). The remission rate was 88.9% at the end of 6 months, with only two reporting no obvious relief of pain.

### 3.4. Sitting Time

**Figure 6:**
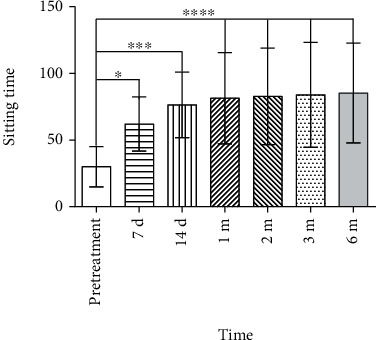
Comparison of sitting time before and after pulsed radiofrequency treatment. The sitting time significantly increased since day 7 after treatment (pretreatment vs. 7 d, ^∗^*P* < 0.05; pretreatment vs. 14 d, ^∗∗∗^*P* < 0.001; and pretreatment vs. 1 m, 2 m, 3 m, or 6 m, ^∗∗∗∗^*P* < 0.0001). There was no significant difference in the sitting time among 7 d, 14 d, 1 m, 2 m, 3 m, and 6 m after treatment (*P* > 0.05).

As many patients were unable to come for each outpatient follow-up due to intervention-irrelevant reasons (e.g., lived too far away from the center), part of the assessments was completed under the guidance of the same physician through telephone interview. Compared with pretreatment (30 ± 15 min), sitting time significantly prolonged on 7 d (62 ± 20 min), 1 m (81 ± 34 min), 2 m (83 ± 36 min), 3 m (84 ± 39 min), and 6 m (85 ± 37 min) after treatment was significantly longer than that before treatment (30 ± 15 min). However, there was no significant difference in the sitting time among 7 d, 14 d, 1 m, 2 m, 3 m, and 6 m after treatment (*P* > 0.05, Figure 6).

### 3.5. Adverse Events

No serious adverse event was reported. No infection occurred following PRF. One patient reported ipsilateral involuntary convulsion of the lower extremity immediately after treatment and recovered within 12 h. Six patients complained of pain at the puncture site, which alleviated shortly with the application of nonsteroidal drugs. Three patients reported mild skin herpes in the perineum within 3 days after treatment and recovered thereafter, which was considered irrelevant to the intervention (Supplement Table 2).

## 4. Discussion

The etiology of PN is complex, and the mechanism is largely unclear. It may be due to the entrapment and mechanical injury of the pudendal nerve. The common entrapment sites are the sciatic foramen and pudendal canal between the sacrotuberous ligament and the sacrospinal ligament, especially the sciatic spine [23, 24]. However, the mechanical injury of the pudendal nerve is often associated with pregnancy, delivery, and pelvic surgery. In addition, the pathological changes of the pudendal nerve may also lead to PN, such as herpes zoster neuralgia and diabetic peripheral neuralgia. In this study, one patient reported herpes zoster neuralgia in the pudendal innervation region, and two patients had diabetic peripheral neuropathy.

For these reasons, conservative treatment (e.g., oral medicine, nerve block therapy, and physical therapy) is usually ineffective. With the continuous development of image-guided technology, interventional treatment has been increasingly performed by clinicians, especially by pain practitioners. Interventional treatment (such as nerve block, radiofrequency, and electrical stimulation treatment) has been applied in the treatment of PN [13, 20], and the efficacy was quite promising.

Radiofrequency technology is a novel technology to treat chronic pain. Traditional continuous radiofrequency technology damages nerves to treat pain in the dominant area, which would inevitably lead to permanent nerve injury and serious side effects. Compared with traditional continuous radiofrequency thermocoagulation technology, pulsed radiofrequency is a nonneurodegenerative radiofrequency technology. The temperature of the electrode tip does not exceed 42°C, which will not cause irreversible tissue damage, in which situation the sensorimotor functions of the nerve could be largely preserved.

However, the underlying mechanism of pulsed radiofrequency in the treatment of neuropathic pain is mostly unknown. Some studies suggested that low-temperature pulsed radiofrequency can form a field effect around the lesion, thus regulating the transmission of pain signals [12]. Compared with continuous radiofrequency thermocoagulation, the PRF procedure shows a similar therapeutic effect and fewer complications [25]. In a typically standard PRF procedure (42°C), the intensity of the electric field is fixed and the efficacy would last for a period of time. In the continuous RF, however, heat effect (as high as 80°C) was used to damage the nerve; thus, the defecation function would be injured as well. Some studies indicated that the electric field effect can inhibit the transmission of excitatory neurotransmitters, activate the immune system, and reduce the chronic inflammatory response [26, 27]. Some researchers suggested that the therapeutic effect of neuropathic pain treatment was based on the electric field [28, 29]. Recently, high-voltage long-duration PRF was reported rewarding for the treatment of chronic pain (e.g., trigeminal neuralgia and postherpetic neuralgia) without obvious nerve injury [15, 30, 31]. It has also been applied in the treatment of PN with the guidance of CT scan [16]. But few studies have reported the therapeutic effect of the ultrasound-guided PRF procedure in PN except for some cases and a small randomized controlled clinical study [18, 19]. It may show a better therapeutic effect than pudendal nerve block only. The application of ultrasound-guided high-voltage long-duration PRF has not been reported in the treatment of PN, as well as anatomical studies and patients' quality of life. Compared with the study reported by Fang et al. [19], we used modified RF parameters. In Fang et al.'s report, the standard radiofrequency ablation mode with 42°C and 120 s pulse consisting of two cycles was used. In our study, a high-voltage (started from 40 V and no more than 80 V) and long-duration (2 Hz of 20 ms width pulses that lasted for 900 s) PRF mode under US guidance was used. As for the outcome measure, VAS and PHQ-9 were used in Fang et al.'s study, while VAS and sitting time were used for the assessment of the efficacy in the present study. According to our clinical experience and patients' reports, sitting time was considered an important subjective index for pain-related quality of life. Studies have shown that sitting time in patients with pudendal neuralgia or chronic pelvic pain will be significantly shortened, and relevant treatment measures can significantly delay the onset time of sitting pain so as to improve the satisfaction of treatment [20, 21, 32]. In a report by Buffenoir et al., sitting time was also used as an evaluation index for pudendal neuralgia [20]. For patients with reduced time of comfortable sitting caused by pain, longer comfortable sitting time not only improved patients' satisfaction with the treatment but also reduced the impact of disease and brought overall well-being and quality of life [21]. The present study indicated that high-voltage long-term PRF treatment can significantly alleviate pain over 6 months and prolong sitting time at least 2 weeks in patients with PN without obvious side effects, suggesting that it can be used as an effective method of PN treatment.

Anatomically, the pudendal nerve goes tortuously and is largely varied. A study showed that before entering the pudendal canal, the branch of the pudendal nerve, the inferior rectum nerve, often runs inward and downward [33]. Even under the guidance of X-ray or CT, there is still the possibility of inaccurate positioning. A report revealed that the pudendal nerve trunk is fixed relative to the surface of the sacrospinous ligament and medial to the ischial spine, and it was adjacent to the internal pudendal artery [22]. It is consistent with the results of our anatomical study (Figures 4(a) and 4(c), Table 3). We further verified the results by cutting off the sacrotuberous ligament (Figure 4(b)). Ultrasound could be a helpful tool to locate the internal pudendal artery and ischial spine in the guidance of the puncturing approach due to its capability of identifying the blood vessel (i.e., internal pudendal artery) and bone landmark (i.e., ischial spine). As a real-time positioning and radiation-free technology, ultrasound-guided PRF avoids vascular damage and is easier and more flexible for operators.

With the internal pudendal artery companied, the pudendal nerve is located on the surface of the sacrospinal ligament. When it goes down into the pudendal canal, its branches are more dispersed, and its relative location to the internal pudendal artery may be varied. Therefore, the pudendal nerve is more accessible in the transverse section of the sciatic spine. Previous studies suggested that the pudendal nerve is adjacent to the sciatic nerve above the cross-section of the sciatic spine, which indicated that the sciatic nerve and even the sacral plexus could be involved if penetrating above the cross-section of the sciatic spine. Therefore, punctuation is recommended to locate at the entrance of the pudendal nerve canal. This provided important information for improving ultrasound-guided treatment for PN [34].

Brusciano et al. reported a novel approach, the dynamic transperineal ultrasound (DTU), in the assessment of the pudendal nerve motility [35, 36]. By this method, pelvic floor neuromuscular integrity such as displacement of puborectalis muscle could be identified. Other pelvic neuromuscular diseases could also be identified, such as ilioinguinal nerve-, genitofemoral nerve-, and obturator nerve-related pelvic pain. In our future study, DTU and electrophysiology examination could be included in the diagnostic process of pudendal neuropathy before application of PRF treatment. The quality of life and pain impact questionnaires also need to be further investigated to evaluate a global response to the intervention in future studies. The sample size of this study was relatively small. A randomized controlled study with a larger sample is needed to draw a final conclusion. High requirement of ultrasound operators also limits the application of this approach. The role and underlying mechanisms of PRF in the treatment of PN still remain to be further explored.

## 5. Conclusions

The present study suggested that high-voltage long-duration PRF treatment can alleviate the pain of patients with pudendal neuralgia and improve their quality of life by prolonging the sitting time without obvious severe adverse events. Ultrasound-guided PRF could be a safe and rewarding treatment for PN patients.

## Figures and Tables

**Table 1:** Diagnostic criteria for pudendal neuralgia (Nantes criteria).

Essential criteria	Complementary diagnostic criteria	Exclusion criteria
Pain in the territory of the pudendal nerve Pain is predominantly experienced while sitting The pain does not wake the patient at night Pain with no objective sensory impairment Pain relieved by diagnostic pudendal nerve block	Burning, shooting, and stabbing pain, numbness Allodynia or hyperpathia Rectal or vaginal foreign body sensation (sympathalgia) Worsening of pain during the day Predominantly unilateral pain Pain triggered by defecation Presence of exquisite tenderness on palpation of the ischial spine Clinical neurophysiology findings in men or nulliparous women	Exclusively coccygeal, gluteal, pubic, or hypogastric pain Pruritus Exclusively paroxysmal pain Imaging abnormalities able to account for the pain

**Table 2:** Distance of the pudendal nerve to the sciatic spine and the pudendal nerve to the internal pudendal artery in the cadavers.

	Distance between the pudendal nerve and the sciatic spine	Distance between the pudendal nerve and the internal pudendal artery
Male	13 mm	6 mm
Female	11 mm	6 mm

**Table 3:** Demographic characteristics of patients.

Characteristic	Content	Value
Numbers (*n*)		20
Sex (*n*)	Male	6
	Female	14
BMI	Range	18.9-31.70
	Mean ± SD	23.7 ± 3.1
Age (years)	Range	42-68
	Mean ± SD	57 ± 8
Pain site	Anus	5
	Genitals	7
	Anus, genitals, and perineum	8
Unilateral and bilateral pain	Unilateral	12
	Bilateral	8
Baseline VAS (points)	Range	5-9
	Mean ± SD	7.0 ± 0.9
Baseline of sitting time (min)	Range	5-52
	Mean ± SD	30 ± 15
Duration of pain	<1 year	5
	1-5 years	10
	>5 years	5
Pain characteristics	Stabbing pain	5
	Burning pain	11
	Aching pain	4
	Throbbing pain	2

## Data Availability

The data used to support the findings of this study are available from the corresponding author upon request.

## Supplementary Materials

Supplementary Materials Supplement Table 1: information of all patients. Supplement Table 2: adverse events. Supplement Table 3: follow-up assessments of all patients.
