# Supplementary material for: Therapeutic Efficacy of Ultrasound-Guided High-Voltage Long-Duration Pulsed Radiofrequency for Pudendal Neuralgia
Source: Neural Plast. 2021 Jul 30;2021:9961145. doi: 10.1155/2021/9961145 (PMC8349273; doi:10.1155/2021/9961145)
Supplement: Supplementary Materials — Supplement Table 1: information of all patients. Supplement Table 2: adverse events. Supplement Table 3: follow-up assessments of all patients. [file 9961145.f1.docx]

**Supplement Table 1. Information of all patients.**

| No. | Gender | Age (yr) | Location of pain | Affected side | Medication before PRF |
| --- | --- | --- | --- | --- | --- |
| 1 | M | 42 | Anus | Bi | Tramadol and Escitalopram oxalate |
| 2 | F | 51 | Genitals | R | Tylox and Pregabalin |
| 3 | F | 64 | Anus, genitals and perineum | L | Fenbid and Pregabalin |
| 4 | F | 68 | Genitals | L | Tramadol and Pregabalin |
| 5 | M | 67 | Genitals and perineum | Bi | Tramadol and Pregabalin |
| 6 | F | 62 | Genitals | L | Tramadol and Pregabalin |
| 7 | M | 53 | Anus and perineum | Bi | Venlafaxine and Celebrex |
| 8 | M | 51 | Anus | Bi | Venlafaxine |
| 9 | F | 43 | Genitals | L | Tramadol and Pregabalin |
| 10 | F | 49 | Genitals | R | Tramadol and Pregabalin |
| 11 | F | 51 | Anus and genitals | Bi | Escitalopram oxalate and Baclofen and Tylox |
| 12 | M | 64 | Genitals | R | Tramadol and Pregabalin |
| 13 | F | 62 | Anus, genitals and perineum | L | Escitalopram oxalate and Pregabalin |
| 14 | M | 64 | Anus | Bi | Escitalopram oxalate and Tramadol |
| 15 | F | 56 | Genitals | L | Escitalopram oxalate and Tramadol and Pregabalin |
| 16 | F | 65 | Anus, genitals and perineum | R | Escitalopram oxalate and Tramadol and Pregabalin |
| 17 | F | 51 | Anus | Bi | Venlafaxine and Celebrex |
| 18 | F | 61 | Anus, genitals and perineum | Bi | Tramadol and Pregabalin |
| 19 | F | 58 | Anus | L | Venlafaxine and Tylox |
| 20 | F | 64 | Anus | R | Venlafaxine and Pregabalin |

**M: male; F: female: Bi: bilateral; R: right; L: left; VAS: visual analogue scale; PRF: pulsed radiofrequency.*

**Supplement Table 2. Adverse events.**

| Event | Timepoints of assessments | | | | | | |  |
| --- | --- | --- | --- | --- | --- | --- | --- | --- |
|  | Baseline | 12h | Day 3 | Day 7 | Month 1 | Month 2 | Month 3 | Month 6 |
| Complications related to PRF treatment – no. of events |  |  |  |  |  |  |  |  |
| Any | 0 | 7 | 3 | 1 | 0 | 0 | 0 | 0 |
| Pain at puncture site | 0 | 6 | 3 | 1 | 0 | 0 | 0 | 0 |
| Convulsion of ipsilateral lower extremity | 0 | 1 | 0 | 0 | 0 | 0 | 0 | 0 |
| Defecation dysfunction | 0 | 0 | 0 | 0 | 0 | 0 | 0 | 0 |
| Sexual dysfunction | 0 | 0 | 0 | 0 | 0 | 0 | 0 | 0 |
| Other complications |  |  |  |  |  |  |  |  |
| Skin herpes | 0 | 0 | 3 | 2 | 0 | 0 | 0 | 0 |

*PRF: pulse radiofrequency.*

**Supplement Table 3. Follow-up assessments of all patients.**

| No. | VAS | | | | | | |  | Sitting time (min) | | | | | | |
| --- | --- | --- | --- | --- | --- | --- | --- | --- | --- | --- | --- | --- | --- | --- | --- |
|  | Pre- | Day 7 | Day 14 | Month 1 | Month 2 | Month 3 | Month 6 |  | Pre- | Day 7 | Day 14 | Month 1 | Month 2 | Month 3 | Month 6 |
| 1 | 7 | 3 | 2 | 3 | 2 | 2 | 2 |  | 27 | 62 | 86 | 72* | 91* | 80* | 93* |
| 2 | 6 | 5 | 4 | 5 | 6 | 6 | 6 |  | 9 | 28 | 45 | 27 | 16 | 13 | 17 |
| 3 | 7 | 2 | 1 | 1 | 1 | 1 | 0 |  | 45 | 77 | 61 | 65* | 66* | 72* | 68* |
| 4 | 7 | 3 | 3 | 5 | 5 | 4 | 5 |  | 18 | 50 | 56 | 37 | 35* | 37* | 40* |
| 5 | 8 | 3 | 2 | 2 | 2 | 2 | 2 |  | 42 | 115 | 127 | 150* | 147* | 180* | 166* |
| 6 | 6 | 3 | 1 | 1 | 1 | 1 | 0 |  | 5 | 43 | 46 | 51 | 55* | 55* | 63* |
| 7 | 7 | 2 | 1 | 0 | 1 | 1 | 1 |  | 46 | 86 | 86 | 99* | 82* | 85* | 79* |
| 8 | 8 | 1 | 0 | 0 | 0 | 0 | 0 |  | 36 | 71 | 92 | 110* | 125* | 115* | 129* |
| 9 | 6 | 4 | 4 | 3 | 3 | 3 | 4 |  | 12 | 75 | 83 | 92 | 96 | 96* | 89* |
| 10 | 7 | 5 | 5 | 5 | 7 | 7 | 7 |  | 47 | 65 | 49 | 60 | 53* | 51 | 46* |
| 11 | 6 | 3 | 4 | 4 | 4 | 4 | 4 |  | 21 | 53 | 59 | 57* | 52 | 61* | 65* |
| 12 | 8 | 3 | 2 | 0 | 0 | 0 | 0 |  | 27 | 51 | 97 | 137* | 140* | 128* | 128* |
| 13 | 9 | 1 | 0 | 2 | 2 | 2 | 2 |  | 45 | 69 | 113 | 97 | 89* | 92* | 93* |
| 14 | 6 | 4 | 2 | 1 | 1 | 1 | 1 |  | 56 | 79 | 98 | 119* | 122* | 125* | 122* |
| 15 | 7 | 8 | 5 | 5 | 5 | 3 | 3 |  | 15 | 58 | 79 | 82* | 96 | 104* | 111* |
| 16 | 7 | 4 | 2 | 2 | 2 | 2 | 2 |  | 39 | 52 | 86 | 93* | 100* | 99* | 100* |
| 17 | 4 | 0 | lost | lost | lost | lost | lost |  | 35 | 60 | lost | lost | lost | lost | lost |
| 18 | 8 | 2 | 0 | 0 | 0 | 0 | 0 |  | 29 | 47 | 71 | 77* | 76* | 76* | 82* |
| 19 | 6 | 2 | 2 | 2 | 2 | 2 | 2 |  | 22 | 36 | 41 | 40 | 49* | 42* | 45* |
| 20 | 8 | 4 | 2 | 2 | lost | lost | lost |  | 18 | 54 | 90* | 95* | lost | lost | lost |

*The star mark * indicated sitting time evaluation conducted through telephone interview under the guidance of the same physician. VAS: visual analogue scale.*
